# The Anatomical Board of Georgia

**Published:** 1888-06

**Authors:** 


### THE ANATOMICAL BOARD OF GEORGIA.

---

The Anatomical Board of the State of Georgia, organized August the 23rd, 1887, held its second meeting in this city on Friday, the 18th inst.

The permanent organization was perfected by adopting a constitution and by-laws and continuing in office, until the regular annual meeting on the fourth Tuesday of next March, the following officers: Thomas R. Wright, M. D., Augusta, President; F. W. McRae, M. D., Atlanta, Secretary; W. S. Elkin, M. D., Atlanta, Treasurer, and an Executive Committee consisting of W. S. Armstrong, M. D., Atlanta, W. P. Nicholson, M. D., Atlanta, G. A. Wilcox, M. D., Augusta, A. G. Thomas, M. D., Atlanta, L. D Carpenter, D. D. S., Atlanta.

The Board is working smoothly and will undoubtedly supply the colleges in this State with an abundance of anatomical material.

The members of the Board are highly gratified with its success thus far.

Since September 5th, 1887, when the first body was received, thirty-one bodies have been received and distributed to the several colleges in proportion to number of matriculants.

The law provides that all bodies which must be buried at public expense, except those claimed by relatives, shall be turned over to the Board. Penalties are also prescribed for failure on the part of officers to comply with the requirements of the law.

Physicians throughout the State can materially aid the cause of medical education in Georgia by lending to the Board their hearty co-operation.

Any officer having in charge any body coming within the jurisdiction of the Board will receive explicit instructions as to its disposition upon notifying the Secretary, Dr. F. W. McRae, No. 63½ Whitehall street, Atlanta, Ga.

The following is the section of the Act referred to:

SEC. 2. That all public officers of this State and their assistants, and all officers and their deputies of every county, city, town or other municipality, and of any and every prison, chain-gang, penitentiary company, morgue, public hospital in this State having charge or control over any dead human body or bodies, not dead from any contagious or infectious diseases, and required to be buried at public expense, are hereby required to notify the said Board of Distributions, or such person or persons as may from time to time be designated in writing by said Board or its duly authorized officers whenever such body or bodies come into his or their possession, charge or control, and shall, without fee or reward, deliver such body or bodies, and suffer the said Board and its duly authorized agents, who may comply with the provisions of this Act, to take and remove all such bodies, to be used only within this State solely for the advancement of medical science; *Provided*, that no such notice shall be given, nor shall any such body or bodies be delivered, if any person claiming to be, and satisfying the authorities in charge of said body or bodies, that he or she is of any degree of kin, or is related by marriage to, or socially or otherwise connected with and interested in the deceased, shall claim the said body or bodies for burial, but it or they shall be at once surrendered to such person for interment, or shall be buried at public expense at the request of such claimant, if a relative by blood or a connection by marriage, provided he or she is financially unable to supply such body or bodies with burial.

*And provided further*, that such notice shall not be given or such body be delivered if the deceased person was a traveler who died suddenly, in which case said body shall be buried;

*And provided further*, that such body or bodies shall in each and every instance be held and kept by the person or persons having charge or control of it or them at least twenty-four (24) hours after death, before delivered to said Board or its agent or agents, during which period notice of the death of such person or persons shall be posted at the court-house door of the county in which said body or bodies are so held.

---

### ITEMS.

THE annual banquet of the Atlanta Society of Medicine will occur at the Markham House Friday night, June 1st.

THE closing exercises of the Training School for Nurses, connected with Spelman Seminary (colored), took place this month.

REMEMBER that ALL communications intended for THE JOURNAL should be addressed to the manager, Dr. A. B. Ashworth, or to the ATLANTA MEDICAL AND SURGICAL JOURNAL, P. O. Box 32, Atlanta, Ga.

DR. FRANK O. STOCKTON, lately professor of Laryngology in the College of Physicians and Surgeons of Chicago, has opened an office at 66½ Whitehall street, Atlanta, Ga., and will limit his practice to the ear, throat and chest.

BOLDIN A NEW HYPNOTIC.—Boldin is the glucoside obtained from boldo leaves, and Dr. Junanville highly praises it, in a recent number of the *Progrès Mèdical*, as a hypnotic "far exceeding opium and chloral." This is saying a good deal for it. We are told also that boldin is not disagreeable to take, has no unpleasant effects, increases the appetite, and has a "strengthening" influence on the patient. Between 5 and 10 grammes were given daily to various patients. The sleep induced by this substance is of a natural kind, and the breathing is regular and tranquil.

Boldo leaves contain about 3 per cent. of boldin. It may be given in capsules in doses of 0.2 grammes (three grains), repeated as necessary, or (diluted 1 in 20 in water) subcutaneously.—*British Medical Journal.*

DR. MILLER B. HUTCHINS (A. M. C., 1886), a prominent young physician formerly of this city, but now of New York, has been appointed Assistant Physician to the Fordham Branch of the New York Skin and Cancer Hospital. From our knowledge of Dr. Hutchins, we know that the position will be well and acceptably filled.

WE have received the prospectus of the *Texas Health Journal*, which will be under the editorial management of Dr. J. R. Briggs, late of the *Courier Record of Medicine*, and published by the *Health Journal* Publishing Co., at Dallas, Tex. The first issue will appear about July 1st and monthly thereafter. Our best wishes to the new comer.

THE MONOPOLY OF ANTIPYRINE.—The French Board of Hospital Charities (Administration de l' Assistance publique) has decided to institute the manufacture of, and supply the hospitals of Paris with, antipyrine, which will henceforth be dispensed under the name *analgesine.*

This move will tend to break the monopoly in this valuable medicine, with whose chemical composition and methods of fabrication manufacturing pharmacists are sufficiently familiar.

The firm that has hitherto had the monopoly of the manufacture and supply of dimethyloxyquinizine, because of the patent on the name *antipyrine*, has become immensely rich out of the large profits accruing from this monopoly. Attention has frequently been directed in these columns to that objectionable feature of antipyrine—that, although its chemical composition was known, it was a patent and proprietary drug.—*Boston Med. and Surg. Journal.*

